# A Radiographic Study of Biomechanical Relationship between the Achilles Tendon and Plantar Fascia

**DOI:** 10.1155/2020/5319640

**Published:** 2020-02-18

**Authors:** Genrui Zhu, Zhifeng Wang, Chengjie Yuan, Xiang Geng, Chao Zhang, Jiazhang Huang, Xu Wang, Xin Ma

**Affiliations:** Department of Orthopedics, Huashan Hospital, Fudan University, No. 12, Middle Wulumuqi Road, Jingan District, Shanghai, China

## Abstract

**Background:**

Previously, scholars have concluded that the Achilles tendon and the plantar fascia were closely biomechanically related, although there is little clinical evidence of the relationship between the two. To investigate the biomechanical relationship between the Achilles tendon and the plantar fascia, the author used standing lateral ankle radiographs of patients with insertional Achilles tendonitis to determine the biomechanical relationship between the Achilles tendon and plantar fascia.

**Methods:**

The author collected standing lateral ankle radiographs from patients with insertional Achilles tendonitis who accepted surgical treatment in the author's hospital from March 2009 to July 2018. According to whether there were bone spurs on the posterior side of the calcaneus, patients were divided into group A (spur present on the posterior side) and group B (spur not present on the posterior side). The positive rates of spurs on the plantar side of the calcaneus were determined in group A and group B. The chi-square test was used to compare the measurement results between the two groups.

**Results:**

In group A, 13 heels were positive for calcaneal bone spurs, and the positive rate was 65.0%. In group B, 3 heels were positive for plantar calcaneal spurs, and the positive rate was 12%. Among all 16 patients with positive plantar calcaneal spurs, 13 had posterior calcaneal spurs (accounting for 81.3%), and 3 had negative results, accounting for 18.7%. There was a significant difference between the results in groups A and B (*P* < 0.05).

**Conclusion:**

There is a relationship between posterior calcaneal spurs and plantar calcaneal spurs in patients with insertional Achilles tendonitis, which can be inferred as resulting from the increasing tension in the biomechanically complex relationship between the Achilles tendon and the plantar fascia.

## 1. Introduction

Heel pain includes pain on the plantar side of the calcaneus and in the posterior heel [1]. Plantar heel pain is often caused by plantar fasciitis, with its typical symptom of pain at the medial calcaneal tubercle that is aggravated after standing for long periods of time or running [2–5]. Bone spurs at the medial calcaneal tubercle is a typical finding on the X-rays of plantar heel pain patients, and they can be the mechanical factors inducing the occurrence of plantar fasciitis [5–7].

Posterior heel pain is often caused by insertional Achilles tendonitis, often combined with the Haglund deformity [8, 9]. The Haglund deformity is an abnormal cystic projection above the posterior calcaneus, which can cause a high pressure state on its rear structure, lead to retrocalcaneal bursitis, and increase the risk of Achilles tendon degeneration and insertional Achilles tendonitis [10]. Further, if the Haglund deformity, insertional Achilles tendonitis and retrocalcaneal bursitis appear, then it is known as Haglund syndrome.

In the current clinical work, it was anecdotally found that patients with plantar heel pain who were diagnosed with plantar fasciitis often presented with posterior heel pain. Due to an anatomical connection between the Achilles tendon, the plantar fascia, and the calcaneal, we proposed a hypothesis that the Achilles tendon, the plantar fascia, and the calcaneal were three parts of a biomechanical complex. And in the high-tension state of this complex, like in the insertional Achilles tendinitis patients, not only the pain appeared on the both sides of calcaneal, but the compensatory bone spurs caused by high tension were found bilaterally also [11, 12].

In the past, the development of bone spurs on both sides of the calcaneus in patients with Achilles tendinopathy and Haglund syndrome has been observed. Fiamengo et al. found that the rate of spurs on the posterior side of the calcaneus in patients with Achilles tendinopathy reached 44% and the rate of plantar spurs in the same population was 6.3% [13]. In a population with Haglund syndrome, the rate of posterior calcaneal spurs was 57% and the rate of plantar spurs was 16% [14]. Menz et al. found that 55% of patients had radiographic evidence of plantar spurs, among whom 56% had an Achilles tendon spur [15]. To further clarify the relationship between plantar spurs and Achilles tendinopathy, Vulcano et al. found that 41.9% of the 785 patients diagnosed with Achilles tendinopathy in outpatient clinics had plantar spurs [16].

Although the rates of bone spurs on the posterior and plantar sides of the calcaneus were observed in previous studies, these studies did not explore the possibility of spurs appeared simultaneously on both sides of calcaneal in the high-tension state of this biomechanically complex.

The aim of this study was to demonstrate the biomechanical relationship between the Achilles tendon and the plantar fascia by comparing the probability of developing bilateral calcaneal spurs with that of developing unilateral calcaneal spurs in the high tension state. So the author retrospectively reviewed patients with insertional Achilles tendinitis who received surgical treatment in the author's hospital and analyzed the bone structure data from standing lateral ankle radiographs.

## 2. Method

The study was a retrospective radiographic review of patients with insertional Achilles tendinitis who were admitted for operations at the orthopedic department in Huashan Hospital of Fudan University (Shanghai, China) from March 2009 to July 2018. The inclusion criteria for the study consisted of patients with a diagnosis of insertional Achilles tendinitis. The diagnosis was made by the senior author based on his clinical examination and radiographic review and was reassured by surgical pathology. All patients had lateral view standing radiographs taken of the ankle.

Patients were excluded from the study group if they had previous Achilles tendon surgery or had noninsertional Achilles tendinitis. The present study was approved by the Institutional Review Board of our institutions, and informed consent was obtained from all participants.

**Figure 1:**
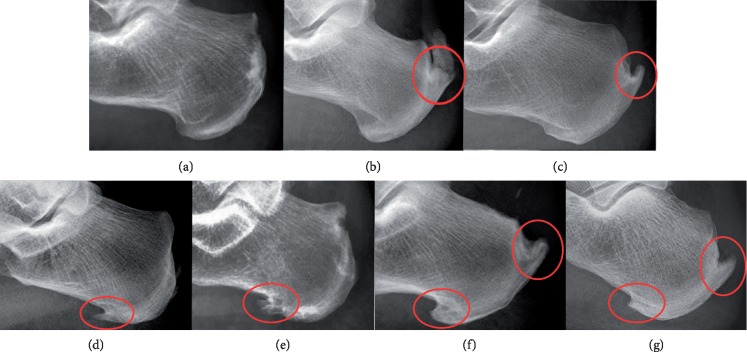
Examples of X-ray obtained in the study. (a) no plantar calcaneal or posterior calcaneal spur; (b, c): posterior calcaneal spurs only; (d, e): plantar calcaneal spurs only; (f, g): spurs on bilateral sides of the calcaneus.

According to whether there were bone spurs on the posterior side of the calcaneus, patients were divided into group A (spur was present on the posterior side) and group B (spur was not present on the posterior side), as shown in Figure 1. Then, the author evaluated and compared the occurrence of plantar calcaneal spurs between groups A and B. All lateral radiographs were reviewed on a picture archiving and communication system by three orthopedic surgeons to identify calcaneal spurs. Patients were considered to have a bone spur only if it was grossly visible without magnification on the standard lateral radiograph.

**Figure 2:**
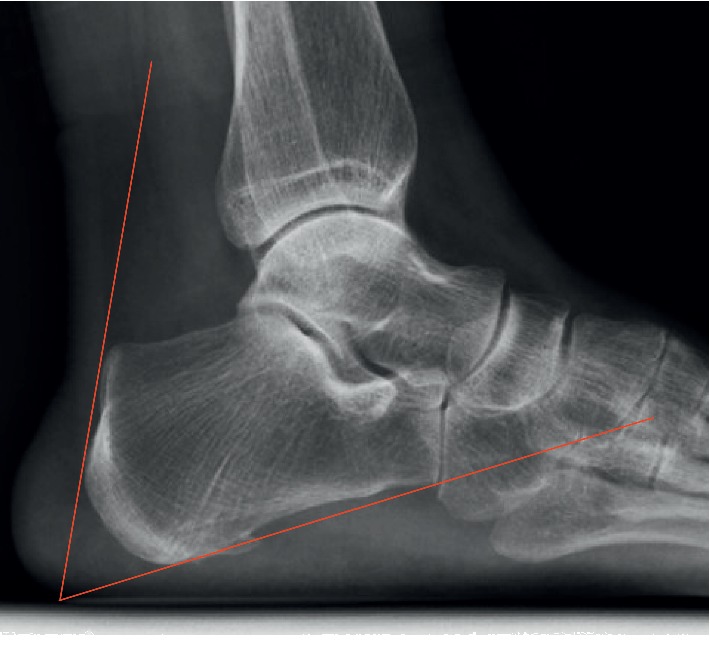
The Fowler–Philip angle was measured between an inferior line which was tangent to the inferior margin of the calcaneocuboid joint and the plantar tuberosity of the calcaneus and a superior line which was tangent to the posterior prominence at the insertion of the Achilles tendon. Normal range is between 44 and 69 degrees, measurements greater than or equal to 75 degrees were thought to be consistent with Haglund's deformity.

**Figure 3:**
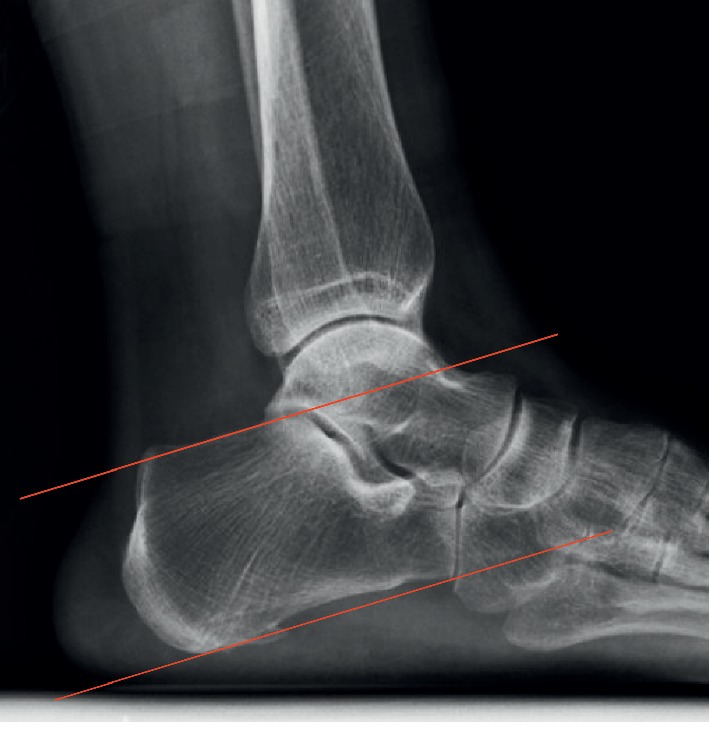
The Parallel patch lines were obtained by first drawing the inferior line from the inferior margin of the calcaneocuboid joint to the plantar tuberosity of the calcaneus. Then the superior line was drawn parallel to the inferior line beginning at the posterior margin of the subtalar joint. If the posterior calcaneal prominence was located above the superior line, it was considered abnormal and consistent with Haglund's deformity.

**Figure 4:**
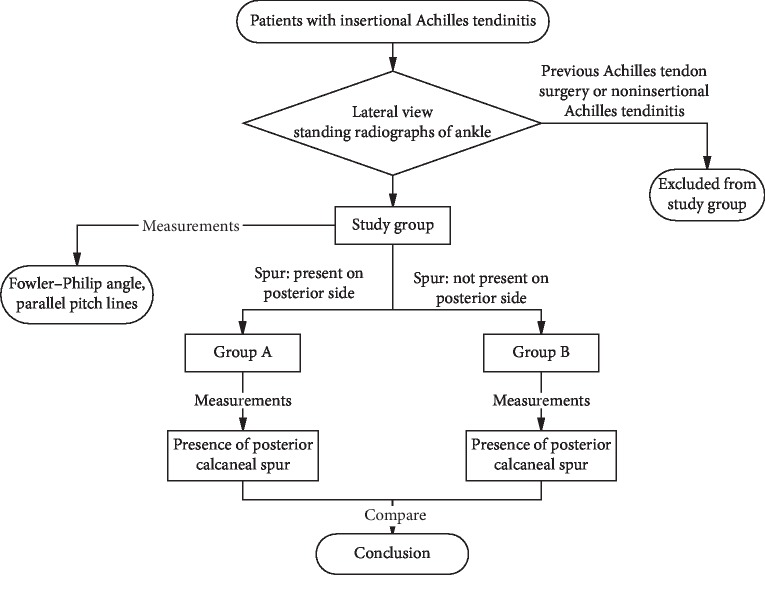
A flowchart to show how the study was conducted.

Since Haglund deformity was believed to have a relationship with insertional tendinitis, the following various measurements were also made: the Fowler-Philip angle, as shown in Figure 2, the parallel pitch lines, as shown in Figure 3, and the presence of a posterior calcaneal spur, as shown in Figure 1 [17–19]. There was a flowchart to show how the study was conducted, as shown in Figure 4.

The chi-square test was applied to compare the measurements between the two groups, with the *P* value set at 0.05 for statistical significance.

## 3. Results

According to the inclusion and exclusion criteria, 43 patients with insertional Achilles tendonitis were enrolled in the study group, with 2 having bilateral Achilles tendonitis, so a total of 45 cases of insertional Achilles tendonitis were collected, as shown in Table 1. Of the 43 patients, 37 were men (86%), and 6 were women (14%), with an average age of 40.7 (range 15 to 69) years. There were 15 left heels (34.9%), 26 right heels (60.5%) and 2 bilateral heels (4.7%).

There were 19 subjects with 20 heels in group A, which consisted of 14 men and 5 women, with an average age of 46.0 ± 12.4 years (range from 17 to 69 years old). There were 24 subjects with 25 heels in group B, which consisted of 23 men and 1 woman, with an average age of 36.9 ± 15.0 years (range from 15 to 65 years old).

**Figure 5:**
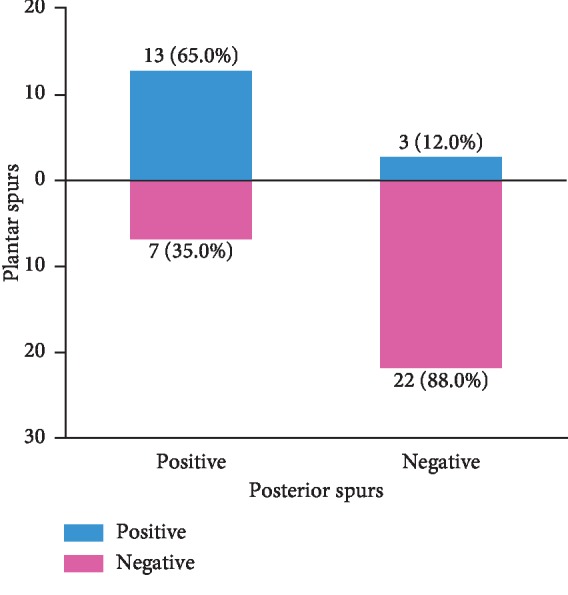
Plantar spurs were present in 65.0% (13 out of 20) of the heels with calcaneal posterior spurs, while there were only 12.0% of heels presenting plantar spurs in the posterior spur negative group. Plantar spurs and posterior spurs were inclined to appear at the same time (65.0%) or would not appear at all (88.0%).

**Figure 6:**
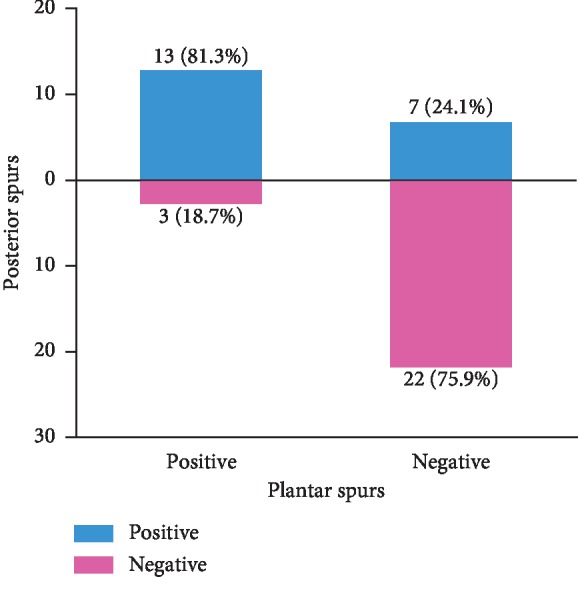
There were 16 out of 45 heels presenting plantar spurs, of which 13 had posterior spurs (81.3%). However, when the plantar spurs were negative, there were only 24.1% of heels presenting posterior spurs.

In group A, calcaneal plantar spurs were present in 65.0% (13 out of 20) of the heels with calcaneal posterior spurs, while in group B, calcaneus plantar spurs were present in 12.0% (3 out of 25), as shown in Figure 5. Calcaneal plantar spurs were found in 16 out of 45 heels, of which 13 had posterior spurs (81.3%), as shown in Figure 6. However, when the plantar spurs were negative (29 out of 45 heels), there were only 24.1% of heels (7 out of 29) presenting posterior spurs, as shown in Table 2. There were significant differences between the two groups (*P* < 0.05). The rate of spurs presenting on both sides of calcaneus was 28.9% (13 out of 45).

In 45 cases of insertional Achilles tendonitis, the positive rate of parallel pitch lines was 75.6% (34 out of 45). The mean Fowler-Philip angle was 51.39 ± 7.85 degrees. There was only one heel in which the Fowler angle was more than 75 degrees (88°).

## 4. Discussion

Pain in the heel includes pain on the plantar side of the calcaneus and posterior heel pain [9, 20, 21]. In the current clinical work, it was anecdotally found that patients with plantar heel pain often presented with posterior heel pain [22]. Calcaneal spurs are outgrowths of bone into tendon and ligamentous attachments, appearing mainly at two points: one at the posterior aspect of the calcaneus near the insertion of the Achilles tendon and the other on the inferior aspect of the calcaneus, coinciding with the insertion of the posterior fibers of the long plantar ligament.

After analyzing imaging studies, the author concluded that there was a relationship between plantar calcaneal spurs and posterior calcaneal spurs in heels with insertional Achilles tendinitis. With regard to plantar calcaneal spurs, group A (posterior spurs were positive) was 5 times more likely than group B (posterior spurs were negative) to have spurs (65% compared to 12%, *P* < 0.05), as shown in Figure 5. Since the mechanical stimulation of plantar calcaneal spurs can lead to plantar fasciitis, which can cause patients to develop heel pain [21], so we thought it was worthwhile for podiatrists to pay attention to calcaneal plantar spurs and the high tension state of the plantar fascia in patients with insertional Achilles tendinitis in the clinical setting. In patients with calcaneal plantar spurs, the positive rate of posterior calcaneal spurs reached 81.3%, which was far greater than that in patients without plantar calcaneal spurs (24.1%), as shown in Figure 6. These results indicated that the probability of developing spurs on both sides of calcaneus was much greater than that of developing unilateral calcaneal spurs. Moreover, an anatomical connection between the Achilles tendon and the plantar fascia makes the relationships among the plantar fascia, calcaneus, and Achilles tendon biomechanically complex, so we assumed this structure may transfer stress from one side of calcaneus to another. Some studies also found that Achilles tendinopathy and heel pain can collectively benefit from similar conservative and surgical treatments by reducing the tension on both sides, such as eccentric calf stretching and gastrocnemius recession [2, 23, 24]. Therefore, the Achilles tendon, calcaneus, and plantar fascia should be considered as a whole entity, and the pain on both sides of calcaneus must be treated simultaneously. Therefore, the other side should receive equal attention when podiatrists encounter pain on either side of calcaneus in clinical work because of this biomechanical complexity.

The Fowler–Philip angle was proposed by Fowler and Philip in 1945, and the normal range is 44–69° [18]. It is believed when the angle is greater than 75°, the Haglund deformity is large enough to cause swelling of the soft tissue at the end of the Achilles tendon, insertional Achilles tendonitis, and other diseases. However, in this survey, the author found that patients with severe insertional Achilles tendonitis whose formal conservative treatment had failed, the average Fowler–Philip angle was 51.39°, and only 1 patient had an angle greater than 70°. This result was inconsistent with Fowler and Philip's theory. Fiamengo et al. also found no difference in the value of the Fowler–Philip angle between the insertional Achilles tendinitis population and a normal population [13, 14, 19]. Therefore, the author does not believe that there is a strong correlation between the Fowler-Philip angle and the occurrence of insertional Achilles tendinitis.

Furthermore, the author found that the positive rate of the parallel pitch line reached 75.6% in the severe cases of insertional Achilles tendonitis requiring surgical treatment, but other studies concluded that the false positive rate of parallel pitch lines in insertional Achilles tendonitis cases ranged from 15% to 56.8% [14, 25, 26]. The author asserts that the heterogeneity of the study populations with regard to the severity of insertional Achilles tendonitis made it infeasible to compare the results of the present study with previous studies. As far as we concerned, different positive rates of parallel pitch lines might be related to different severities of insertional Achilles tendinitis, but this concept needs further study.

The limitations of this study are as follows: first, in this study, the author only studied the correlation between spurs on two sides of the calcaneus in insertional Achilles tendonitis patients who received surgical treatment, and there was no case of insertional Achilles tendonitis that could be alleviated by conservative treatment included as a control group. Therefore, future studies need to explore the correlation between spurs on the two sides of the calcaneus in patients with less severe Achilles tendinitis who only need conservative treatment. Second, the purpose of the study was to deduce the biomechanically complex relationships among the Achilles tendon, calcaneus, and plantar fascia via radiography; the results suggest the necessity of jointly considering these two diseases in clinical practice, but the two diseases were not directly investigated in this study. For this reason, future research can directly target the correlation between the two diseases with regard to pathogenesis, treatment, and prognosis. Last, the study group only enrolled 43 patients which was a relatively small sample size. Furthermore, there was only 1 female patient in group B, and there were 5 female patients in group B. As far as we concerned, the main reason was because there were only 24 patients enrolled in group B and 19 patients in group A, so it was the small number of patients that caused sampling error.

## 5. Conclusion

First, through weight-bearing lateral ankle X-rays, a relationship between posterior calcaneus spurs and plantar calcaneus spurs in insertional Achilles tendinitis patients was found, and a biomechanical complex involving the Achilles tendon, calcaneus, and plantar fascia was deduced. It was suggested that insertional Achilles tendonitis and plantar fasciitis should be considered to be related and treated synthetically in clinical practice. Second, the positive rate of the parallel pitch lines was higher in insertional Achilles tendonitis patients, and the Fowler–Philip angle was not predictive of insertional Achilles tendonitis.

## Figures and Tables

**Table 1:** Patient profiles in group A and group B.

Patient group	Patient numbers				Heel numbers			
	Male	Female	Total	Average age (years)	Left	Right	Bilateral	Total
Group A	14	5	19	46 ± 12.4	5	14	1	20
Group B	23	1	24	36.9 ± 15.0	10	12	1	23
Total	37	6	43	40.9 ± 14.5	15	26	2	43

**Table 2:** The presence of calcaneal plantar spurs in group A and group B. Positive means that calcaneal plantar spur is observed on the radiograph; negative means that calcaneal plantar spur is not observed on the radiograph.

	Positive	Negative	Total
Group A	13	7	20
Group B	3	22	25
Total	16	29	45

## Data Availability

The datasets generated during and/or analyzed during the current study are available from the corresponding author on reasonable request.
